# Metabolic Remapping at “Point‐Line‐Plane” Levels With Central Dysfunction in Cerebral Small Vessel Disease

**DOI:** 10.1002/brb3.70773

**Published:** 2025-08-22

**Authors:** Jie Ma, Juan‐Juan Lu, Xin Gao, Jia‐Jia Wu, Xiang‐Xin Xing, Mou‐Xiong Zheng, Xu‐Yun Hua, Jian‐Guang Xu

**Affiliations:** ^1^ Department of Rehabilitation Medicine, Yueyang Hospital of Integrated Traditional Chinese and Western Medicine Shanghai University of Traditional Chinese Medicine Shanghai China; ^2^ School of Rehabilitation Science Shanghai University of Traditional Chinese Medicine Shanghai China; ^3^ Universal Medical Imaging Diagnostic Center Shanghai China; ^4^ Department of Traumatology and Orthopedics, Shuguang Hospital Shanghai University of Traditional Chinese Medicine Shanghai China; ^5^ Engineering Research Center of Traditional Chinese Medicine Intelligent Rehabilitation Ministry of Education Shanghai China

**Keywords:** brain networks, cerebral small vessel disease, glucose metabolism, metabolic connectivity

## Abstract

**Background:**

This study is aimed at investigate the glucose metabolic patterns of cerebral small vessel disease (CSVD) at “point‐line‐plane” levels.

**Methods:**

We retrospectively collected the ^18^F‐fluorodeoxyglucose PET/MRI images of 174 CSVD patients and 206 healthy controls. First, the brain FDG‐PET of each subject was divided into 7 classical networks, and the mean standard uptake value (SUV_mean_) was calculated. Second, the SUV_mean_ of different brain networks was compared at the region of interest and voxel level. Third, the metabolic connectivity of intra‐ and inter‐networks was compared. Fourth, the correlation of SUV_mean_ changes in different networks was calculated. Finally, the correlation between the SUV_mean_ of the whole network and each brain region within the network was analyzed in different brain networks.

**Results:**

Increased SUV_mean_ was mainly shown in the right postcentral gyrus in the somatomotor network and the right superior parietal gyrus in the dorsal attention network; decreased SUV_mean_ was predominantly observed in the right superior temporal gyrus in the somatomotor network in the CSVD group. Significant correlations of SUV_mean_ alterations in varying networks and SUV_mean_ of the whole network and each brain region within the network were found.

**Conclusion:**

Affected by CSVD, the glucose metabolism redistribution is heterogeneous in different brain network, and its changes start from brain region and metabolic connectivity within the sub‐brain network.

## Graphical abstract

The glucose metabolism redistribution is heterogeneous in different network, its changes start from brain regions and connectivity within the sub‐brain network in cerebral small vessel disease.

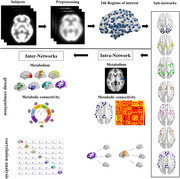

## 1 Introduction

Cerebral small vessel disease (CSVD) is frequently detected in older adults through magnetic resonance imaging (MRI), manifesting as white matter lesions, recent small subcortical infarcts, lacunes, enlarged perivascular spaces, and microbleeds (Wardlaw et al. 2013). Consequences or manifestations of CSVD vary among patients, including cognitive disorders such as dementia, as well as non‐cognitive disorders such as abnormalities of gait and balance (De Silva and Faraci 2020). Actually, CSVD without a clear‐cut symptomatology is not an innocuous bystander, which has also contributed to the development of disabling conditions such as dementia and stroke (Pasi and Cordonnier 2020). Therefore, CSVD has become a great concern for researchers.

By measuring the cerebral metabolic rate of glucose, fluorodeoxyglucose positron emission tomography (FDG‐PET) can identify local regions showing hypometabolism in patients with CSVD (Heiss 2018). However, there is no report on the glucose metabolic changes in different brain networks in CSVD. Increased evidence shows that the brain network is crucial in disease performance and process, and CSVD disrupted its topological characteristics and intrinsic between‐ and within‐network connectivity, especially the dorsal attention, default, and frontoparietal networks, underlining the significance of brain network connectivity disruption for the expression of the clinical symptoms of the disease (Liu et al. 2019; Schulz et al. 2021; Li et al. 2023). The reason for the functional network disruption induced by CSVD remains unclear. Glucose is the primary energy source for normal brain functions, particularly in processes such as signal transduction and neurotransmission (Shulman et al. 2004). Its metabolic pattern is fairly consistent with brain function activity from the newborn (Chugani 1998). Therefore, we hypothesized that abnormal glucose metabolism also existed in the brain networks in CSVD.

The unique potentials of FDG‐PET in localizing and quantifying metabolic changes make this technique play an important role in understanding the mechanisms by which CSVD causes consequences. Physiologically, this technique is grounded in the coupling between synaptic activity and glucose metabolism, more directly reflecting neuronal activity (Magistretti 2006). Metabolic connectivity, calculated from interregional correlations in FDG uptake, provides a stable measure of brain activity that is less susceptible to transient fluctuations and is relatively independent of neurovascular coupling (Horowitz et al. 2025). It exhibited a linear association with regional glucose metabolic rates, indicating a significant coupling between temporal metabolic connectivity and cerebral energy demands (Tomasi et al. 2017). However, most radiology studies on brain networks use fine‐grained scientific research data, and few real‐world PET studies identified brain metabolic characteristics in CSVD patients, especially metabolic network changes. A large amount of medical data is generated every day in the real world. The availability of real‐world medical data, as well as data mining and analysis, has triggered more and more real‐world evidence research and promoted personalized health care, which has important health economics significance (Frieden 2017). Therefore, we retrospectively analyzed real‐world FDG‐PET data that included 174 patients with CSVD and 206 healthy individuals. Here we reported inter‐ and intra‐changes in glucose metabolism and metabolic connectivity in seven common networks to provide new insights into the abnormal brain network induced by CSVD from the perspective of nuclear medicine.

## 2 Material and Methods

### 2.1 Participants

A total of 380 subjects, who visited our center from January 2016 to December 2019 and underwent ^18^F‐fluorodeoxyglucose PET/MRI ([18F]FDG‐PET/MRI) scanning, were retrospectively collected in the case‐control study, including 174 patients with CSVD and 206 healthy controls matched by age and sex.

Inclusion criteria (Castello et al. 2021) for CSVD: 1) age ≥ 40 yr; 2) independent in daily living (Modified Rankin Scale (Bruno et al. 2010) ≤ 1); 3) the National Institutes of Health Stroke Scale (NIHSS) (Kwah and Diong 2014) score = 0; 4) Clinical Dementia Rating (CDR) score (Cedarbaum et al. 2013) = 0; 5) punctate or early confluent or confluent subcortical white matter hyperintensities (WMHs) (Wardlaw et al. 2013), confirmed by axial FLAIR sequences; 6) no other abnormal signal in brain MRI (e.g., lacunar, perivascular space, hemorrhage, infarct, malacia, space‐occupying lesions).

Inclusion criteria for healthy controls: (1) age ≥ 40 yr; 2) independent in daily living (Modified Rankin Scale ≤ 1); (3) no stroke symptoms, as assessed by the NIHSS; (4) Clinical Dementia Rating (CDR) score = 0; (5) no abnormal signal in brain MRI.

Participants with other neurological disorders (e.g., stroke, dementia), severe systemic conditions (e.g., cancer, significant metabolic disorders), or psychiatric conditions were excluded.

Three experts—two experienced clinicians and one radiologist—independently made the diagnosis, while Siemens PET data post‐processing software (molecular imaging neuroimaging) was concurrently used to further support the diagnostic process.

Description of vascular risk factors: (1) smoking: more than one cigarette per day for 6 consecutive or cumulative months. (2) hypertension, diabetes, and hyperlipidemia: diagnosed for more than 1 year, and the condition was stable after regular medication.

### 2.2 Image Acquisition

PET scanning was performed on a Biograph mMR (Siemens Healthcare, Erlangen, Germany). Prior to the scan, participants were instructed to fast for at least 6 h and ensure that their blood glucose levels were within the normal range (≤8.3 mmol/L). The injection dose of [18F]FDG was 3.7 MBq/kg. During the uptake period, participants rested quietly in a dimly lit room while remaining awake. Subsequently, a 10‐minute scanning was performed across five bed positions covering the head to mid‐thigh region (3 min per bed position). During the scanning, participants were asked to close their eyes and remain still. Reconstruction of PET data was obtained using the ordered subsets expectation maximization algorithm with 3 iterations, 21 subsets, and a 4.0‐mm Gaussian filtering (slice thickness = 2.03 mm, matrix size = 172 × 172, in‐plane resolution = 4.17 mm × 4.17 mm).

### 2.3 Data Preprocessing

**FIGURE 1:**
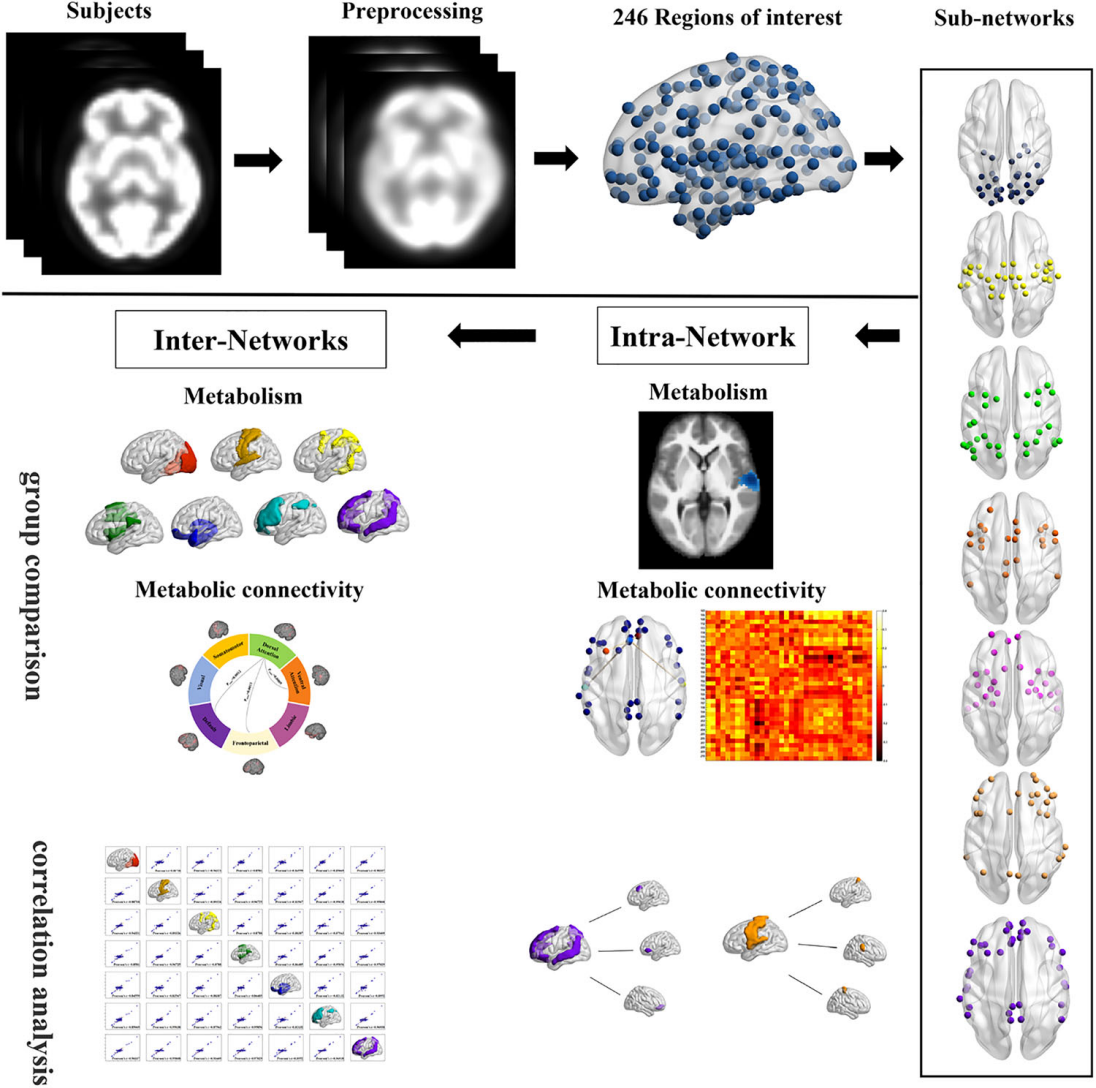
**Flowchart of analysis**. Preprocessed PET data were divided into seven classic networks (Yeo et al. 2011) based on the human Brainnetome Atlas with 246 brain regions (BNA‐246) (Fan et al. 2016). The metabolism, metabolic connectivity, and correlation of mean standard uptake value were analyzed in different networks.

The pipeline of our framework was outlined in Figure 1. Processing was performed by the Statistical Parametric Mapping 12.0 toolbox (SPM12; http://www.fil.ion.ucl.ac.uk/spm/) based on the Matlab 2013b platform. The preprocessing approaches were briefly as follows: (1) Transform images into NIFTI format. (2) Crop edges to preserve the brain part. (3) Check PET and T1 image quality. (4) Reset the origin manually to the anterior commissure, including PET and T1 images. (5) Perform space normalization to the Montreal Neurology Institute (MNI) space through T1 images. Specifically, T1 images were first spatially normalized to the MNI standard space. The resulting transformation parameters were then applied to the PET images to achieve their spatial normalization into the MNI space. (6) Conduct smoothing with a Gaussian kernel of 10 mm full width at half maximum.

### 2.4 Definition of Regions of Interest (ROI)

A total of 246 brain regions defined by the human Brainnetome Atlas were included as ROIs in intra‐network analysis (Fan et al. 2016). Seven classical sub‐networks (visual, somatomotor, dorsal attention, ventral attention, limbic, frontoparietal, and default networks) were defined as ROIs in inter‐network analysis (Yeo et al. 2011). The average standard uptake value (SUVmean) within the ROIs for each participant was normalized by global SUVmean to correct for variability in the injection (Huo et al. 2019). The global SUVmean was calculated as the average uptake across all voxels within a standard whole‐brain binary mask in MNI space. The SUV_mean_ of each ROI was extracted for inter‐group comparison of metabolism and correlation analysis.

### 2.5 Construction and Analysis of Metabolic Connectivity

In the metabolic network, the inter‐subject covariance of SUV_mean_ was calculated using Pearson's correlation coefficients (r) to describe metabolic connectivity (edges) between each pair of ROIs (nodes). Seven correlation matrixes, each including all ROI pairs in the corresponding network, were constructed for a group of subjects, forming the weighted, undirected metabolic network. Inter‐group comparisons of metabolic connectivity were statistically analyzed with a non‐parametric permutation test, with 10000 permutations.

### 2.6 Statistical Analysis

Clinical and PET data were analyzed using SPSS 21.0 and SPM12. We compared baseline characteristics of patients and controls with a two‐sample *t*‐test for continuous data and χ^2^ test for categorical data. SUV_mean_ and metabolic connectivity between two groups were analyzed with a two‐sample *t* test. Pearson correlation was applied to analyze the relationship of glucose metabolism in the different networks. The significance level was set at a two‐tailed p‐value of less than 0.05. Effect size is determined with Cohen's D (Tipton et al. 2017) (small = 0.2, medium = 0.5, large = 0.8). The False Discovery Rate (FDR) method was applied for multiple comparison correction in PET data.

## 3 Results

### 3.1 Inter‐Group Comparison

#### 3.1.1 Demographic Characteristics and Vascular Risk Factors

A total of 174 CSVD patients (101 males) and 206 healthy controls (132 males) were finally collected. No significant intergroup differences were observed in age, gender, BMI, FBS, smoking, diabetes, and hyperlipidemia (p > 0.05), whereas a significant difference was found in hypertension (p = 0.016) (Table 1).

**TABLE 1:** Demographic characteristics and vascular risk factors of CSVD patients and healthy controls.

Characteristics	CSVD group (n = 174) M(SD)	HC group (n = 206) M(SD)	p‐value
Demographic characteristics			
Age—year	53.75 (7.78)	53 (18.42)	0.613^a^
Males—No. (%)	101 (58.05)	132 (64.08)	0.246^b^
BMI—kg/m^2^	24.22 (3.19)	24.02 (3.08)	0.540^a^
FBS	5.78 (1.60)	5.55 (1.20)	0.104^a^
**Vascular risk factors**			
Smoking—No. (%)	28 (16.09)	35 (16.99)	0.890^b^
Hypertension—No. (%)	52 (29.89)	39 (18.93)	**0.016** ^b^
Diabetes—No. (%)	30 (17.24)	26 (12.62)	0.245^b^
Hyperlipidemia—No. (%)	44 (25.29)	58 (28.16)	0.562^b^

Abbreviations: CSVD: cerebral small vessel disease; HC: healthy control; FBS: fasting blood sugar; M: mean; SD: standard deviation.

^a^ two sample t‐test.

^b^ Chi‐square test.

#### 3.1.2 Comparison of FDG‐SUV in Different Networks

**FIGURE 2:**
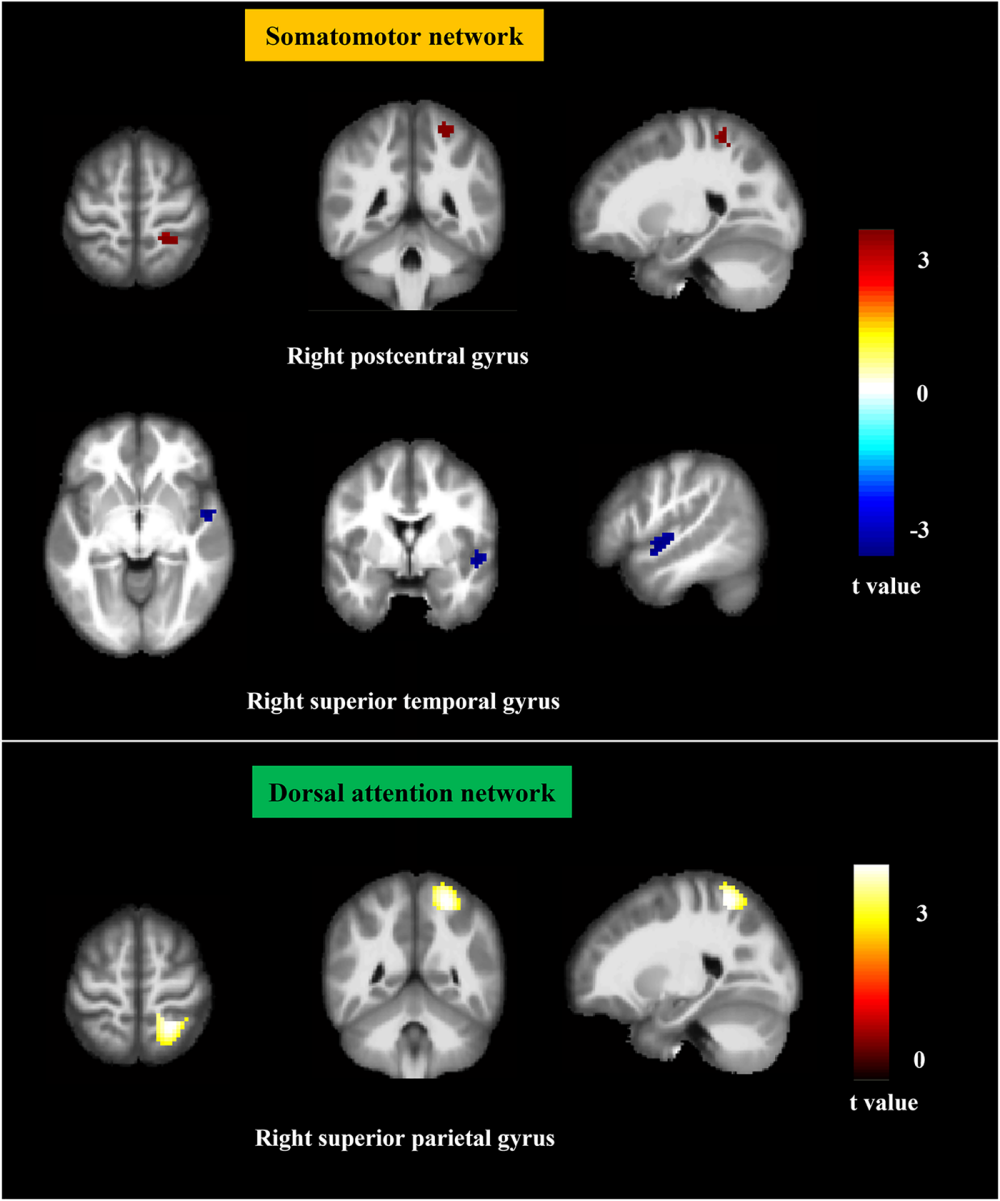
**Comparison of glucose metabolism in different functional networks**. Positive and negative t‐values represented higher and lower metabolism in patients than in healthy controls, respectively.

As for glucose metabolism, ROI‐based and voxel‐based SUV_mean_ of different networks were compared. No statistical difference was found in the two groups when each network is compared as a whole ROI (p > 0.05). Table 2 showed the significant different brain regions after comparing the SUV_mean_ in each network (p < 0.05). After voxel‐based analysis in each network, CSVD patients exhibited increased FDG‐SUV values in the right postcentral gyrus in the somatomotor network and right superior parietal gyrus in the dorsal attention network (p < 0.05, FDR corrected), as well as decreased FDG‐SUV values in the right superior temporal gyrus in the somatomotor network (p < 0.05, FDR corrected). (Figure 2, Table 3). No statistical difference was found in other networks.

**TABLE 2:** The SUV_mean_ of different networks in CSVD patients and healthy controls.

Network name	CSVD group (n = 174) M(SD)	HC group (n = 206) M(SD)	p value	Cohen's D
**Visual network**	1.155(0.036)	1.155(0.135)	0.968	0.00
Right fusiform gyrus (lateroventral area)	1.144(0.052)	1.156(0.052)	0.032	0.23
Left posterior parahippocampal gyrus	1.048(0.055)	1.059(0.050)	0.036	0.21
Right caudal lingual gyrus	0.972(0.053)	0.984(0.053)	0.033	0.23
Right middle occipital gyrus	1.052(0.061)	1.040(0.053)	0.037	0.21
Right occipital polar cortex	1.059(0.070)	1.045(0.056)	0.035	0.22
Left inferior occipital gyrus	1.154(0.051)	1.166(0.044)	0.019	0.25
Left lateral superior occipital gyrus	1.194(0.066)	1.176(0.064)	0.007	0.28
**Somatomotor network**	1.074(0.034)	1.076(0.127)	0.896	0.02
Left superior frontal gyrus (medial area)	1.096(0.056)	1.108(0.050)	0.036	0.23
Right precentral gyrus (head and face region)	1.350(0.088)	1.325(0.084)	0.005	0.29
Right precentral gyrus (upper limb region)	1.222(0.068)	1.208(0.064)	0.035	0.21
Right superior temporal gyrus (area 41/42)	1.028(0.047)	1.038(0.043)	0.033	0.22
Left superior temporal gyrus (TE1.0 and TE1.2)	1.126(0.051)	1.141(0.044)	0.002	0.32
Left superior parietal lobule (postcentral area)	0.840(0.055)	0.851(0.043)	0.038	0.23
Right superior parietal lobule (postcentral area)	0.864(0.051)	0.875(0.043)	0.030	0.23
Right inferior parietal lobule (rostroventral area)	1.228(0.085)	1.211(0.068)	0.035	0.22
Left postcentral gyrus (upper limb, head, and face region)	0.992(0.085)	1.010(0.074)	0.029	0.23
Right postcentral gyrus (area 2)	0.958(0.081)	0.976(0.066)	0.019	0.25
Left postcentral gyrus (trunk region)	0.837(0.094)	0.862(0.079)	0.007	0.29
Right postcentral gyrus (trunk region)	0.862(0.079)	0.814(0.083)	0.010	**0.59**
**Dorsal attention network**	0.993(0.029)	0.994(0.119)	0.983	0.01
Left middle frontal gyrus (ventrolateral area)	0.919(0.618)	0.937(0.056)	0.003	0.04
Right inferior frontal gyrus (dorsal area)	1.109(0.070)	1.080(0.064)	0.000	0.43
Left precentral gyrus (caudal dorsolateral area)	1.301(0.076)	1.280(0.076)	0.007	0.28
Right precentral gyrus (caudal dorsolateral area)	1.228(0.071)	1.209(0.067)	0.007	0.28
Right inferior temporal gyrus (ventrolateral area)	1.146(0.069)	1.161(0.056)	0.027	0.24
Left superior parietal lobule (caudal area)	0.832(0.060)	0.844(0.046)	0.028	0.23
Left postcentral gyrus (area 2)	0.959(0.076)	0.975(0.063)	0.029	0.23
**Ventral attention network**	1.123(0.036)	1.126(0.133)	0.786	0.03
Left inferior frontal gyrus (opercular area)	1.090(0.068)	1.063(0.060)	0.000	0.42
Left inferior frontal gyrus (ventral area)	1.125(0.084)	1.101(0.069)	0.003	0.31
Left paracentral lobule (lower limb region)	1.142(0.052)	1.156(0.053)	0.013	0.27
Left caudoposterior superior temporal sulcus	1.184(0.069)	1.159(0.056)	0.000	0.40
Right caudoposterior superior temporal sulcus	1.202(0.066)	1.189(0.053)	0.036	0.22
Right dorsal agranular insula	0.887(0.047)	0.897(0.042)	0.021	0.23
Right ventral dysgranular and granular insula	1.147(0.074)	1.124(0.059)	0.001	0.35
Left cingulate gyrus (caudodorsal area)	1.169(0.062)	1.183(0.051)	0.019	0.25
Right cingulate gyrus (caudodorsal area)	1.151(0.053)	1.162(0.046)	0.024	0.22
**Limbic network**	0.951(0.031)	0.967(0.114)	0.056	0.18
Left orbital gyrus (area 13)	1.013(0.053)	0.999(0.044)	0.004	0.29
Right orbital gyrus (area 13)	1.130(0.067)	1.115(0.052)	0.021	0.25
Right superior temporal gyrus (medial area)	1.115(0.067)	1.096(0.058)	0.004	0.31
Left parahippocampal gyrus (entorhinal cortex)	1.052(0.076)	1.035(0.053)	0.015	0.26
**Frontoparietal network**	1.076(0.043)	1.076(0.129)	0.928	0.00
Left superior frontal gyrus (medial area)	1.246(0.097)	1.226(0.085)	0.039	0.22
Right middle frontal gyrus (dorsal area)	0.907(0.056)	0.919(0.052)	0.038	0.22
Left middle frontal gyrus (dorsolateral area)	0.970(0.054)	0.982(0.050)	0.026	0.23
Right middle frontal gyrus (dorsolateral area)	0.936(0.055)	0.949(0.048)	0.019	0.25
Right middle frontal gyrus (area 46)	0.784(0.061)	0.799(0.054)	0.012	0.26
Left inferior frontal sulcus	1.079(0.065)	1.063(0.055)	0.011	0.27
Right inferior frontal sulcus	1.176(0.081)	1.149(0.067)	0.001	0.37
Right orbital gyrus (lateral area)	1.010(0.055)	1.023(0.042)	0.010	0.27
Left inferior temporal gyrus (caudolateral area)	1.074(0.057)	1.088(0.051)	0.011	0.26
**Default network**	1.075(0.025)	1.079(0.125)	0.609	0.04
Left middle frontal gyrus (ventrolateral area)	0.912(0.064)	0.927(0.061)	0.023	0.24
Left inferior frontal gyrus (caudal area)	1.157(0.075)	1.138(0.066)	0.009	0.27
Right inferior frontal gyrus (caudal area)	1.050(0.068)	1.031(0.062)	0.005	0.29
Left inferior frontal gyrus (rostral area)	1.080(0.062)	1.092(0.051)	0.043	0.21
Left orbital gyrus (medial area)	1.118(0.064)	1.102(0.052)	0.009	0.28
Right orbital gyrus (medial area)	1.077(0.049)	1.067(0.045)	0.041	0.21
Left orbital gyrus (orbital area)	1.233(0.091)	1.216(0.069)	0.035	0.21
Left superior temporal gyrus (rostral area)	1.184(0.083)	1.168(0.068)	0.034	0.21
Left anterior superior temporal sulcus	0.915(0.083)	0.931(0.070)	0.038	0.21
Right rostroposterior superior temporal sulcus	1.160(0.065)	1.142(0.052)	0.005	0.31
Right precuneus (area 31)	0.861(0.079)	0.878(0.066)	0.026	0.24

Abbreviations: CSVD: cerebral small vessel disease; HC: healthy control; M: Mean; SD: standard deviation; SUV_mean_: mean standard uptake value.

^a^ Cohen's D effect size interpretation (Tipton et al. 2017) (small = 0.2, medium = 0.5, large = 0.8)

^b^ two sample t‐test.

**TABLE 3:** Brain region information of glucose metabolic comparison between CSVD patients and healthy controls.

Brain regions	Cluster size	Cluster centroid MNI coordinates			t value
		x	y	z	
Somatomotor network					
right postcentral gyrus	25	24	−45	63	3.719
right superior temporal gyrus	58	51	0	−6	−3.780
Dorsal attention network					
right superior parietal gyrus	174	24	−51	63	3.876

Abbreviations: CSVD, cerebral small vessel disease; MNI, montreal neurological institute.

#### 3.1.3 Comparison of Metabolic Connectivity

**FIGURE 3:**
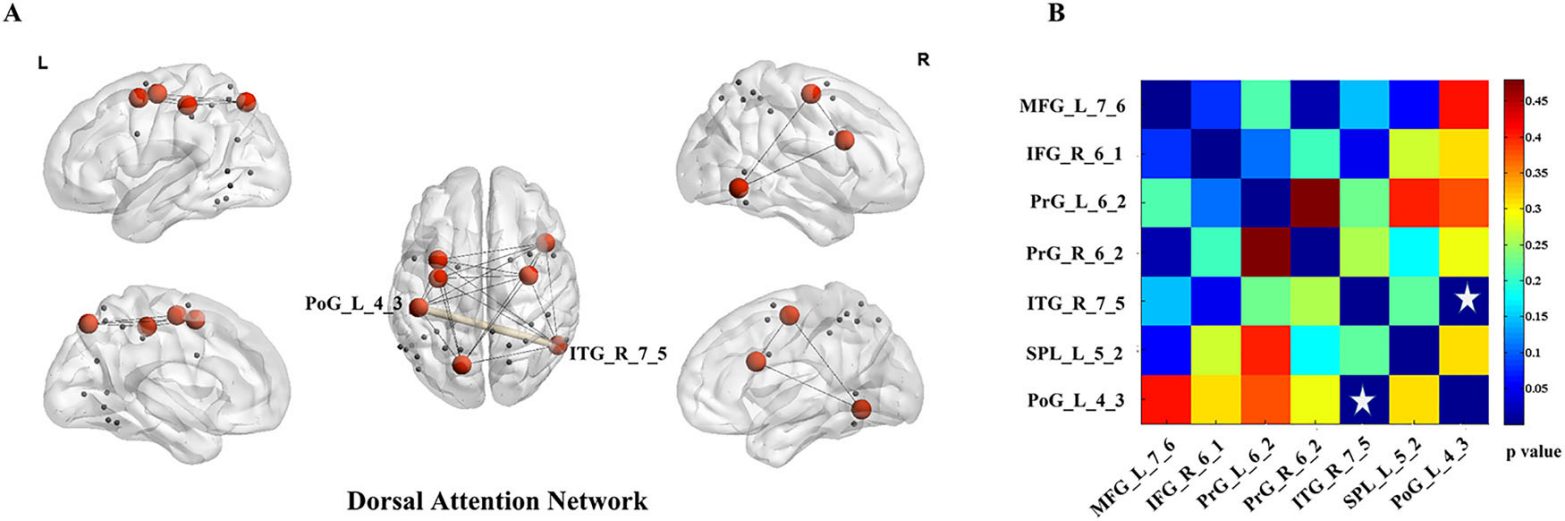
**Inter‐group differences of the metabolic connections in the dorsal attention network**. A shows nodes and edges of the dorsal attention network. The red nodes are brain regions with statistical differences in glucose metabolism. B shows the statistical chart after comparison of metabolic connectivity. A white five‐pointed star indicates the statistically significant connections (p < 0.05, FDR corrected). Abbreviations: PoG_L_4_3: Left postcentral gyrus (area 2); ITG_R_7_5: Right inferior temporal gyrus (ventrolateral area); MFG_L_7_6: Left middle frontal gyrus (ventrolateral area); IFG_R_6_1: Right inferior frontal gyrus (dorsal area); PrG_L_6_2: Left precentral gyrus (caudal dorsolateral area); PrG_R_6_2: Right precentral gyrus (caudal dorsolateral area); SPL_L_5_2: Left superior parietal lobule (caudal area).

We compared inter‐group differences of the metabolic connections among seven networks and found no significant change of metabolic connectivity among these networks (p > 0.05). Figure 3 showed the results of metabolic connectivity in intra‐network; a significant statistical difference was found in metabolic connectivity between the right inferior temporal gyrus (ventrolateral area) and the left postcentral gyrus (area 2) in the dorsal attention network (p < 0.05, FDR corrected).

### 3.2 Correlation Analysis

#### 3.2.1 Inter‐Network Correlation Analysis

**FIGURE 4:**
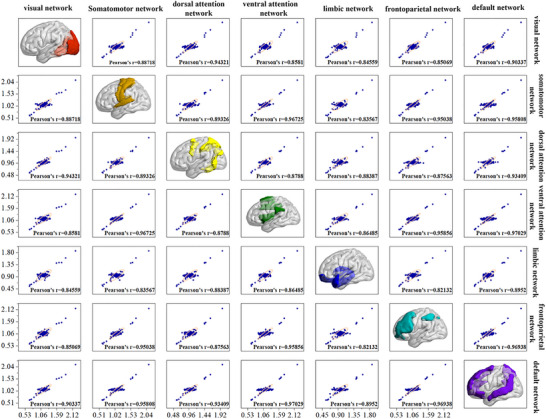
**Correlation analysis of mean standard uptake value (SUV_mean_) of different networks**. The Pearson correlation analysis showed significant associations among the SUV_mean_ of visual, somatomotor, dorsal attention, ventral attention, limbic, frontoparietal and default networks (p < 0.001).

The Pearson correlation analysis showed significant associations among the SUV_mean_ of visual, somatomotor, dorsal attention, ventral attention, limbic, frontoparietal, and default networks (p < 0.001) (Figure 4).

#### 3.2.2 Intra‐Network Correlation Analysis

**FIGURE 5:**
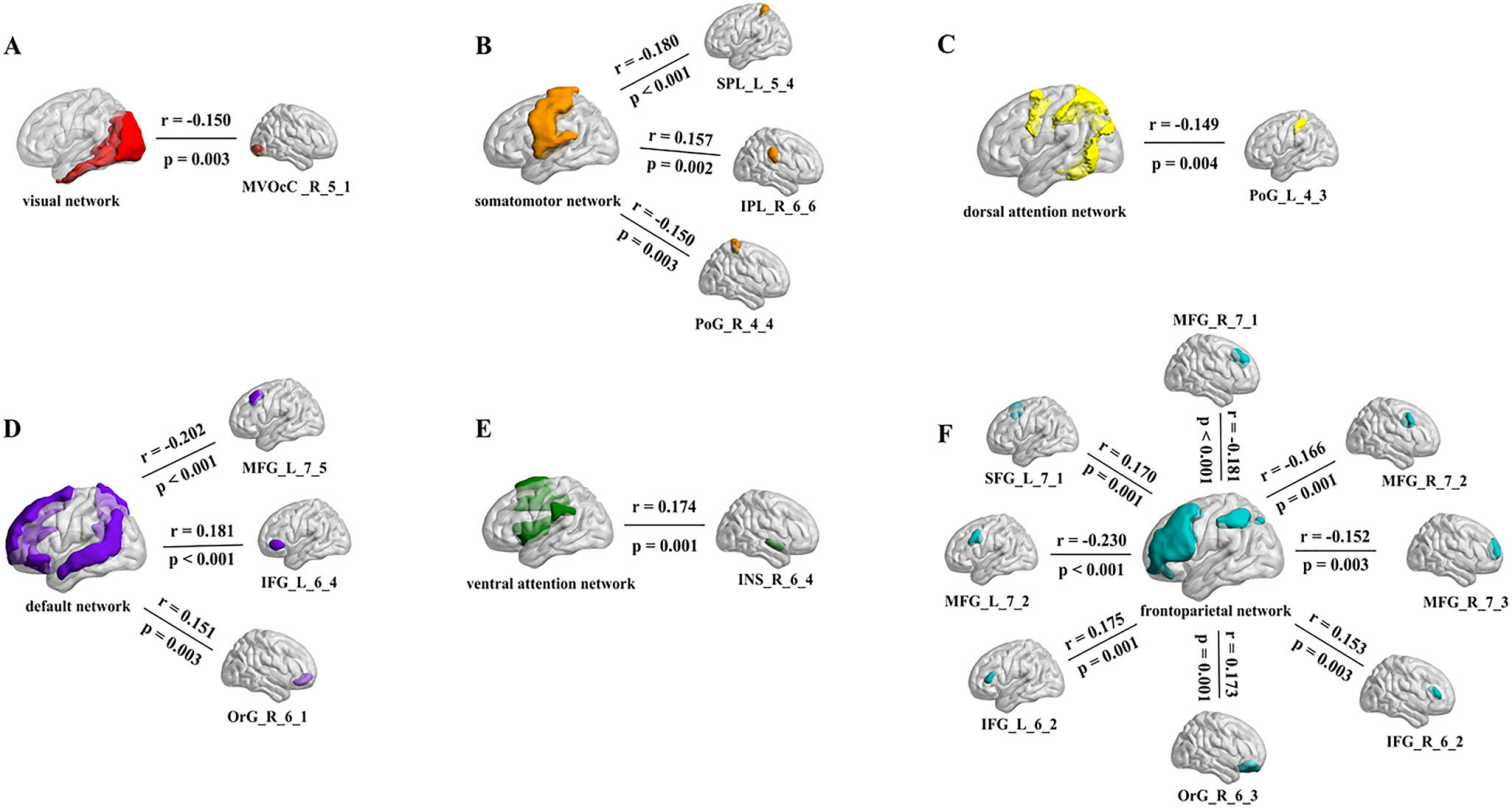
**intra‐network correlation analysis**. Significant correlations were found in SUV_mean_ of the whole network and each brain region within the network (p < 0.05). Abbreviations: MVOcC_R_5_1: Right caudal lingual gyrus; SPL_L_5_4: Left superior parietal lobule (postcentral area); IPL_R_6_6: Right inferior parietal lobule (rostroventral area); PoG_R_4_4: Right postcentral gyrus (trunk region); PoG_L_4_3: Left postcentral gyrus (area 2); MFG_L_7_5: Left middle frontal gyrus (ventrolateral area); IFG_L_6_4: Left inferior frontal gyrus (rostral area); OrG_R_6_1: Right orbital gyrus (medial area); INS_R_6_4: Right ventral dysgranular and granular insula; MFG_R_7_1: Right middle frontal gyrus (dorsal area); SFG_L_7_1: Left superior frontal gyrus (medial area); MFG_L_7_2: Left middle frontal gyrus (inferior frontal junction); IFG_L_6_2: Left inferior frontal sulcus; OrG_R_6_3: Right orbital gyrus (lateral area); IFG_R_6_2: Right inferior frontal sulcus; MFG_R_7_3: Right middle frontal gyrus (area 46); MFG_R_7_2: Right middle frontal gyrus (inferior frontal junction).

Significant correlations were observed between the SUV_mean_ in visual network and the SUV_mean_ in right caudal lingual gyrus (r = ‐0.150, p = 0.003); between the SUV_mean_ in somatomotor network and the SUV_mean_ in left superior parietal lobule (postcentral area) (r = ‐0.180, p < 0.001), right inferior parietal lobule (rostroventral area) (r = 0.157, p = 0.002), and right postcentral gyrus (trunk region) (r = ‐0.150, p = 0.003); between the SUV_mean_ in dorsal attention network and the SUV_mean_ in left postcentral gyrus (area 2) (r = ‐0.149, p = 0.004); between the SUV_mean_ in default network and the SUV_mean_ in left middle frontal gyrus (ventrolateral area) (r = ‐0.202, p < 0.001), left inferior frontal gyrus (rostral area) (r = ‐0.181, p < 0.001), and right orbital gyrus (medial area) (r = 0.151, p = 0.003); between the SUV_mean_ in ventral attention network and the SUV_mean_ in right ventral dysgranular and granular insula (r = 0.174, p = 0.001); between the SUV_mean_ in frontoparietal network and the SUV_mean_ in right middle frontal gyrus (dorsal area) (r = ‐0.181, p < 0.001), left superior frontal gyrus (medial area) (r = 0.170, p = 0.001), left middle frontal gyrus (inferior frontal junction) (r = ‐0.230, p < 0.001), left inferior frontal sulcus (r = 0.175, p = 0.001), right orbital gyrus (lateral area) (r = 0.173, p = 0.001), right inferior frontal sulcus (r = 0.153, p = 0.003), right middle frontal gyrus (area 46) (r = ‐0.152, p = 0.003), and right middle frontal gyrus (inferior frontal junction) (r = ‐0.166, p = 0.001) (Figure 5).

## 4 Discussion

In this study, we investigated the abnormal glucose metabolism and metabolic connectivity in seven classical networks derived from real‐world FDG‐PET data in CSVD. As a main finding, we revealed that the glucose metabolism redistribution is heterogeneous in different networks; its changes start from brain region and metabolic connectivity within the sub‐brain network in CSVD, which may be the characteristic metabolic pattern of the brain network; and account for functional alterations in the brain networks in CSVD. This finding sheds light on the metabolic disruptions within brain networks associated with CSVD and underscores a potential linkage between its clinical manifestations and alterations in glucose metabolism.

[18F]FDG‐PET imaging displays the local cerebral glucose consumption, which directly reflects the level of neuronal activity (Berti et al. 2014). Hypometabolism on FDG‐PET is thought to reflect the cumulative loss of neuropil and impaired synaptic/neuronal function. Compared to other neuroimaging techniques, FDG‐PET provides a unique perspective by examining brain alterations from a metabolic standpoint. It enables the quantification of cerebral glucose consumption, which is directly linked to synaptic activity (Attwell and Iadecola 2002). This makes FDG‐PET a valuable tool for capturing subtle metabolic changes that may precede functional and structural abnormalities. A series of studies has highlighted the utility of [18F]FDG‐PET in advancing the understanding of many neurological diseases (Berti et al. 2014; Mosconi et al. 2008; Silverman et al. 2001). As metabolic variations occur at the level of synaptic connections (Berti et al. 2014), [18F]FDG‐PET is a feasible technique for investigating metabolic changes in the brain. Our findings suggested decreased synaptic/neuronal function in certain functional networks may be related with pathological conditions of CSVD. It was consistent to the animal experiments and proton magnetic resonance spectroscopy studies (Wang et al. 2012; Balbi et al. 2019; Ping et al. 2019; Di et al. 2012).

In order to explore metabolic changes in specific networks, the brain was divided into seven classical networks based on different functions (Yeo et al. 2011). These networks provided reference for functional segregation of the brain. The brain is organized with functionally and anatomically distinct cortical and subcortical structures. To thoroughly elucidate the cerebral structural and functional foundations underlying diseases, a reliable brain atlas is essential, as it can accurately reflect the subdivisions of brain regions. However, many brain atlases lack detailed parcellation and fail to provide comprehensive information, as they do not integrate both structural and functional information. In the present study, we chose the human brainnetome atlas, which is composed of 210 cortical and 36 subcortical subregions, offering information on both anatomical and functional connectivity (Fan et al. 2016). We compared glucose metabolism in each functional network separately and found regions with changed metabolism mainly located in the right postcentral gyrus, superior parietal gyrus, and superior temporal gyrus, which were closely related to somatosensory and cognitive function. This finding may reveal why CSVD contributes to abnormalities of gait and balance and dementia.

Specifically, in addition to participating in basic attentional processes, the superior parietal gyrus was suggested to be associated with executive function and short‐term storage (Wager and Smith 2003; Otsuka et al. 2006). The enhanced superior parietal gyrus activity in the left hemisphere is related to executive function, whereas the right is related to short‐term storage (Otsuka et al. 2008). The maintenance of visuospatial attention and short‐term memory storage in CSVD patients may be supported by increased metabolism in the right rostral superior parietal gyrus. We also found metabolic abnormalities in the superior temporal gyrus, which contributes to the process of semantic priming (Laufer et al. 2011). The semantic priming effect refers to the promotion of the threshold priming word on the lexical confirmation of the subsequent target word with semantic association, which consists of different processes: automatic processing and control processing. Previous researche demonstrated deficits in executive/attention function and semantic/phonemic fluency in CSVD (Herbert et al. 2014). The right postcentral gyrus mediates short‐term memory tasks and proprioception (Peters et al. 2009). Our findings provide support for a past study that found that functional abnormalities of the right posterior central gyrus in CSVD (Feng et al. 2021).

Metabolic connectivity and functional connectivity are commonly used metrics to characterize interregional brain connectivity. Functional connectivity, derived from functional magnetic resonance imaging (fMRI), refers to the temporal synchrony of blood‐oxygen‐level‐dependent (BOLD) signals between different brain regions. It provides an indirect measure of neural activity and is influenced by neurovascular coupling (Di and Biswal 2012). In contrast, metabolic connectivity reflects the correlations in glucose metabolism across brain regions, offering a more stable representation of inter‐regional coordination associated with neural activity (Choi et al. 2014; Lee et al. 2008). An interesting finding was that the dorsal attention network is the only one that shows both metabolic and metabolic connectivity abnormalities, which is engaged in attentional processes (Dumais et al. 2018; Fox et al. 2005). Specifically, it modulated externally oriented attention, such as top‐down guided voluntary activation (Hopfinger et al. 2000). For example, one previous study has identified that this network activates during cognitive tasks requiring the involvement of external attention (Costumero et al. 2015). Although our study only investigated resting brain state related with CSVD, it was similar to previous conclusions, and attentional regions were particularly affected. The attention/executive domain is the cognitive domain most commonly implicated in CSVD, often assessed by tasks such as phonemic and semantic fluency (Salvadori et al. 2020). Our results were consistent with their findings and might suggest that executive/attention function and semantic priming were the earliest impaired cognitive functions. It was noted that only intra‐network metabolic connectivity changes were observed in our study, while inter‐network connectivity remained largely stable. Previous studies have suggested that the extent of brain network disruptions is associated with the overall severity and total burden of CSVD (Feng et al. 2024; Tuladhar et al. 2016). As the burden of CSVD increases, functional connectivity tends to decline more markedly (Li et al. 2021). These lines of evidence suggest that the disease severity among the CSVD patients included in the present study may not have reached a threshold sufficient to induce widespread alterations in intra‐ or inter‐network metabolic connectivity. Additionally, the brain atlas may also be a potential contributing factor, as different atlases involve distinct parcellation schemes, which may be based on structural features, functional characteristics, or an integration of both. However, given that the atlas employed in our study integrates both structural and functional information, the resulting findings are considered robust. In particular, the observed alterations in intra‐network metabolic connectivity among the included CSVD patients are deemed reliable, supporting the validity of our results within this population.

This study has several limitations. Firstly, the CSVD patients included in this study tended to be younger in age, with lower proportions of smoking, hypertension, diabetes, and hyperlipidemia. Moreover, all participants were from a Chinese population. These factors may limit the generalizability of our findings to broader CSVD populations. Future studies should aim to validate these findings in more heterogeneous and demographically diverse CSVD cohorts. Secondly, this study did not assess clinical characteristics; therefore, it was not possible to establish a direct association between region‐specific metabolic abnormalities and functional decline. In future work, we aim to incorporate comprehensive clinical functional assessments in CSVD patients to provide more robust metabolic evidence underlying functional impairments. Thirdly, our study calculated metabolic connectivity changes based on PET imaging at the group level, which may lose individual internal information and not fully represent connectivity. Our future study will adopt a new method to calculate the metabolic connectivity within individuals, which may reflect the information of individuals. Lastly, future studies employing a longitudinal design may better establish the causal relationship between network metabolism and clinical outcomes in CSVD.

## 5 Conclusions

In summary, we explored the change of glucose metabolism based on large‐sample brain FDG‐PET data in CSVD patients and calculated the metabolic connectivity between subjects in the brain networks, which showed functional abnormalities in previous related studies as well. We found that the glucose metabolism redistribution is heterogeneous in different networks; its changes start from the brain region and metabolic connectivity within the sub‐brain network in CSVD. In the future, we will further explore the glucose metabolism characteristics of different cerebrovascular risk factors, subtypes, and different clinical manifestations in CSVD.

## Author Contributions

**Jie Ma**: writing – original draft, writing – review and editing, funding acquisition, data curation. **Juan‐Juan Lu**: writing – original draft, formal analysis, visualization. **Xin Gao**: data curation, formal analysis, investigation, validation. **Jia‐Jia Wu**: methodology, investigation, funding acquisition. **Xiang‐Xin Xing**: data curation, formal analysis. **Mou‐Xiong Zheng**: conceptualization, supervision. **Xu‐Yun Hua**: conceptualization, supervision, funding acquisition. **Jian‐Guang Xu**: conceptualization, funding acquisition, project administration.

## Ethics Statement

This retrospective study was approved by the institutional review board of our hospital (No. 2020–188).

## Conflicts of Interest

The authors declare no conflicts of interest.

## Peer Review

The peer review history for this article is available at https://publons.com/publon/10.1002/brb3.70773.

## Data Availability

The data supporting this study's findings are available from the corresponding author upon reasonable request.
